# Combination Therapy of Prostate Cancer by Oncolytic Adenovirus Harboring Interleukin 24 and Ionizing Radiation

**DOI:** 10.3389/fonc.2020.00421

**Published:** 2020-04-03

**Authors:** Li-jun Mao, Yi Kan, Bing-heng Li, Sai Ma, Yirui Liu, Dong-liang Yang, Chunhua Yang

**Affiliations:** ^1^Department of Urology, Affiliated Hospital of Xuzhou Medical University, Xuzhou, China; ^2^Jiangsu Key Laboratory of Biological Cancer Therapy, Xuzhou Medical University, Xuzhou, China; ^3^Department of Radiotherapy, Affiliated Hospital of Xuzhou Medical University, Xuzhou, China

**Keywords:** IL-24, oncolytic adenovirus, ionizing radiation, prostate cancer, combined treatment

## Abstract

Prostate cancer is a common malignant tumor and the second leading cause of cancer-related death in men. Radiation therapy is a curative treatment for localized prostate cancer and has a limited effect for castration-resistant prostate cancer (CRPC). Interleukin 24 (IL-24) has a radiosensitizing effect in cancer cells. Our previous studies showed that ZD55-IL-24, an oncolytic adenovirus harboring IL-24, had better anti-tumor effect with no toxicity to normal cells. In this study, we evaluated the synergistic anti-tumor effect of oncolytic adenovirus ZD55-IL-24 combined with radiotherapy in prostate cancer. *In Vitro* and *In Vivo* experiments showed that the combined therapy significantly inhibited the growth of prostate cancer and provoked apoptosis of prostate cancer cells. In conclusion, the combination of ionizing radiation and oncolytic adenovirus expressing IL24 could achieve synergistic anti-tumor effect on prostate cancer, and is a promising strategy for prostate cancer therapy.

## Introduction

Prostate cancer is a common cancer in the male (1). Prostatectomy, radiation therapy, chemotherapy, and androgen deprivation therapy are main methods for the treatment of prostate cancer (2). For early stage prostate cancer, most patients show tumor regression and reduced prostate specific antigen (PSA) level after treatment. However, after long-term androgen deprivation therapy, castration-resistant prostate cancer (CRPC) will develop, leading to poor prognosis (3). In particular, CRPC shows chemoresistance and radioresistance (4, 5). Therefore, novel treatment strategy for CRPC is urgently required.

Interleukin 24 (IL-24) is originally produced in human melanoma tumor cells and exhibits anti-tumor effect by enhancing cancer cell apoptosis, inhibiting cancer metastasis, and improving immune regulation (6). IL-24 can be used to treat various cancers but has no obvious adverse effects on normal cells (7). Oncolytic adenovirus is a natural or genetically modified viral species that selectively infects and kills tumor cells (8). Cancer targeting gene-viral therapy (CTGVT), which has better anti-tumor effects than gene therapy alone or viral therapy alone, was designed by inserting a tumor suppressor gene into an oncolytic viral vector (9). Previous study showed that oncolytic adenovirus ZD55-IL-24 with the deletion of E1B-55 gene and the insertion of IL-24 gene, had a better anti-tumor effect than ZD55 with no toxicity to normal cells (10). In this study, we evaluated the synergistic anti-tumor effect of oncolytic adenovirus ZD55-IL-24 combined with radiotherapy on prostate cancer. We further explored the underlying mechanisms to provide potential strategies for clinical treatment of prostate cancer.

## Materials and Methods

### Cell Culture

PC-3 cell, DU-145 cell line and HEK-293 cell lines were provided by the Institute of Biochemistry of Chinese Academy of Sciences, cultured in RPMI-1640 or DMEM medium supplemented with 10% fetal bovine serum (Gibco, MA, USA) and 100 U penicillin/ streptomycin (Gibco), and maintained at 37°C in a humid incubator with 5% CO_2_. Cells were irradiated with different doses of X-ray by Varian Clinac 23EX Linear Accelerator (Varian, USA).

### Recombinant Adenovirus

ZD55-IL-24 was provided by the Institute of Biochemistry of the Chinese Academy of Sciences and the construct was described previously (10). Mass production of the virus was performed in HEK293 cells using the Adeno-XTM Maxi Purification Kit (Clontech, USA). Viral titers were determined with the QuickTiterTM Adenovirus Titer Immunoassay Kit (Cell Biolabs, San Diego, CA, USA).

### CCK-8 Assay

Cell Counting kit-8 (CCK-8; Dojindo Molecular Technologies, Inc., Japan) was used to evaluate cell proliferation. PC-3 and DU-145 cells were seeded in 96-well plates with 3,000 cells per well. Then cells were exposed in different doses of X-ray (0, 2, 5, 10, 15 GY); or treated with ZD55-IL-24 of different titers (0, 1, 10, 20, 50 MOI); and treated with PBS, 10 GY X-ray, 10 MOI ZD55-IL-24, 5 MOI ZD55-IL-24 plus 5 GY X-ray. The radiation was performed at 12 h after virus injection. After 24, 48, 72 and 96 h incubation, 10 μL CCK-8 solution was added into each well. With 1-4 h incubation, the absorbance of cells at 450 nm was measured by microplate reader.

### Hoechst33258 Staining

The apoptosis of cells was assessed by Hoechst33258 staining. PC-3 and DU-145 cells were seeded in 6-well plates. Cells were treated with PBS, 10 GY X-ray, 10 MOI ZD55-IL24, 5 MOI ZD55-IL-24 plus 5 GY X-ray. The radiation was performed at 12 h after virus infection. After 48 h incubation, cells were fixed by 4% paraformaldehyde. Next the cells were stained with Hoechst33258 for 10 min, washed with PBS 3 times, and observed under fluorescence microscope.

### TUNEL Assay

In situ apoptosis assay kit (KeyGenBio, Nanjing, China) was used to stain apoptotic cells. The nuclei were counterstained with DAPI. Apoptotic cells were observed under a fluorescence microscope and cells in 6 randomly selected fields were counted.

### Western Blot Analysis

Proteins were extracted from cells and tumor tissues using protein extraction kit. Equal amounts of protein were separated by SDS-PAGE and transferred to PVDF membranes. The membranes were then incubated with antibodies for IL-24 (1:1000, Proteintech, USA), Caspase-3 (1:1000, Proteintech, USA), Caspase-8 (1:1000, Proteintech, USA), Bcl-2 (1:1000, Abcam, UK) and β-actin (1:1000, Proteintech, USA). After incubation at 4°C overnight, the membranes were incubated with the secondary antibody for 2 h. Protein bands were then detected using ECL reagents and the gray scale values of the bands were analyzed using Image-J software.

### Xenograft Tumor Model

Five-week old male BALB/c nude mice were obtained from Vital River Laboratory Animal Technology (Beijing, China). All animal experiments were approved by Institution Committee on Animal Care and Use. A xenograft model was established by subcutaneous injection of 1 × 10^6^ PC-3 cells into each mouse. Nude mice were divided into 4 groups (*n* = 5): (1) PBS group: mice received intratumoral injection of PBS; (2) Radiation group: mice were exposed by 10 GY X-ray in 10th day; (3) ZD55-IL-24 group: mice received intratumoral injection of ZD55-IL24 (1 × 10^9^ pfu) every 3 days; (4) Combination group: mice received intratumoral injection of ZD55-IL24 (5 × 10^8^ pfu) every 3 days and exposed by 5 GY X-ray in 10th day; Tumor volume was measured on 7 days after subcutaneous injection of cells. Tumor volume was then measured every 3 days. Until the 28th day, all the mice were killed. Tumor volume (TV) was calculated by the following formula: TV (mm^3^) = length × width^2^ × 0.5.

### Hematoxylin-Eosin Staining

Tissues of xenograft tumors were dissected and fixed with 10% formalin. After embedding in paraffin, the tissue sample was cut into a thickness of 5 μm. After deparaffinization, the sections were stained with hematoxylin-eosin and fixed with a neutral resin. The morphology and pathological changes of the samples were observed with an optical microscope (Nikon DS-Ri1, Japan), and 5 randomly selected non-repetitive regions were photographed.

### Immunohistochemical Staining

The sample in the paraffin was cut into a thickness of 4 μm. After deparaffinization and hydration, the sections were subjected to antigen retrieval. The endogenous peroxidase activity of the sections was blocked with 3% hydrogen peroxide. After incubation with blocking serum for 30 min, the sections were incubated with antibodies for Caspase-3 (1:500, Proteintech, USA), Caspase-8 (1:500, Proteintech, USA) and Bcl-2 (1:500, Abcam, Uk). After incubation at 4°C overnight, sections were stained with DBA kit (ZSGB-Bio, Beijing, China). The sections were observed under optical microscope (Nikon DS-Ri1, Japan) and analyzed by Image-J software.

### Statistical Analysis

The data were analyzed by SPSS 16.0 and plotted by Graphpad Prism 6 software. Data were expressed as mean ± SD. The T test was used for comparison between the two groups. One-way ANOVA was used for comparison among multiple groups. *P* < 0.05 was considered significant.

## Results

### Inhibition of Prostate Cancer Cell Proliferation by ZD55-IL24 and Radiation

**Figure 1:**
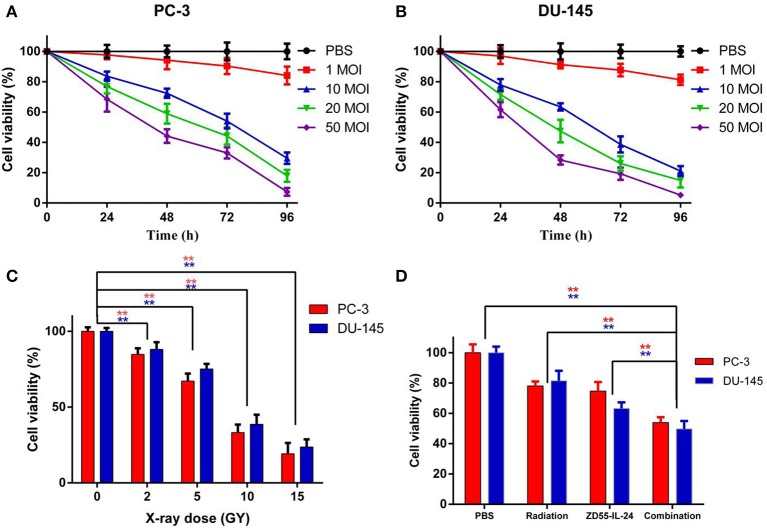
The proliferation of PC-3 and DU-145 cells exposed to ZD55-IL-24 or/and radiation. **(A)** Different titers of ZD55-IL-24 inhibited the proliferation of PC-3 cells. **(B)** Different titers of ZD55-IL-24 inhibited the proliferation of DU-145 cells. **(C)** Different doses of radiation inhibited the proliferation of PC-3 and DU-145 cells. **P < 0.01 vs. PBS group. **(D)** The combination of ZD55-IL-24 and radiation inhibited the proliferation of PC-3 and DU-145 cells after 48 h. **P < 0.01 vs. combination group.

ZD55-IL-24 inhibited the proliferation of PC-3 and DU-145 cells in a time and dose dependent manner (Figures 1A,B, *P* < 0.01). Radiation inhibited the proliferation of PC-3 and DU-145 cells in a dose dependent manner (Figure 1C, *P* < 0.01). We chose 5 MOI ZD55-IL-24 plus 5 GY X-ray as combination treatment. After 48 h of treatment, cell proliferation in combination group was significantly lower than that of the ZD55-IL-24 group or radiation group (Figure 1D, *P* < 0.01).

### Induction of Prostate Cancer Cell Apoptosis by ZD55-IL24 and Radiation

**Figure 2:**
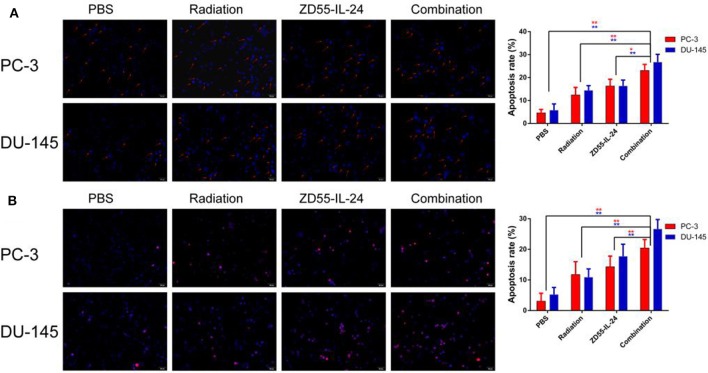
The apoptosis of PC-3 and DU-145 cells exposed to ZD55-IL-24 or/and radiation. **(A)** Hoechst-33258 staining showed the apoptosis rate of PC-3 and DU-145 cells in each group (200×). **(B)** TUNEL assay showed the apoptosis rate of PC-3 and DU-145 cells in each group (200×). **P < 0.01 vs. combination group.

Hoechst-33258 staining showed that the apoptosis rate of combination group, ZD55-IL-24 group, radiation group and PBS group in PC-3 cells was (20.54 ± 3.11)%, (15.52 ± 2.34)%, (13.72 ± 3.65)%, (5.75 ± 1.60)%, respectively, with significant difference between the combination treatment group and the monotherapy group (Figure 2A, *P* < 0.01). The apoptosis rate of combination group, ZD55-IL-24 group, radiation group and PBS group in DU-145 cells was (24.92 ± 3.37)%, (17.59 ± 2.26)%, (11.36 ± 3.56)%, (4.81 ± 2.83)%, respectively, with significant difference between the combination treatment group and the monotherapy group (Figure 2A, *P* < 0.01).

In addition, TUNEL assay showed that the apoptosis rate of combination group, ZD55-IL-24 group, radiation group and PBS group in PC-3 cells was (20.44 ± 2.57)%, (14.31 ± 3.47)%, (11.76 ± 4.20)%, (3.06 ± 2.57)%, respectively, with significant difference between the combination treatment group and the monotherapy group (Figure 2B, *P* < 0.01). The apoptosis rate of combination group, ZD55-IL-24 group, radiation group and PBS group in DU-145 cells was (26.65 ± 3.08)%, (17.71 ± 3.98)%, (10.90 ± 2.71)%, (5.23 ± 2.30)%, respectively, with significant difference between the combination treatment group and the monotherapy group (Figure 2B, *P* < 0.01). Taken together, these data indicated that ZD55-IL-24 combined with radiation had better apoptosis-inducing capability than single therapy.

### The Expression of Apoptosis Related Proteins in Prostate Cancer Cells Treated by ZD55-IL24 and Radiation

**Figure 3:**
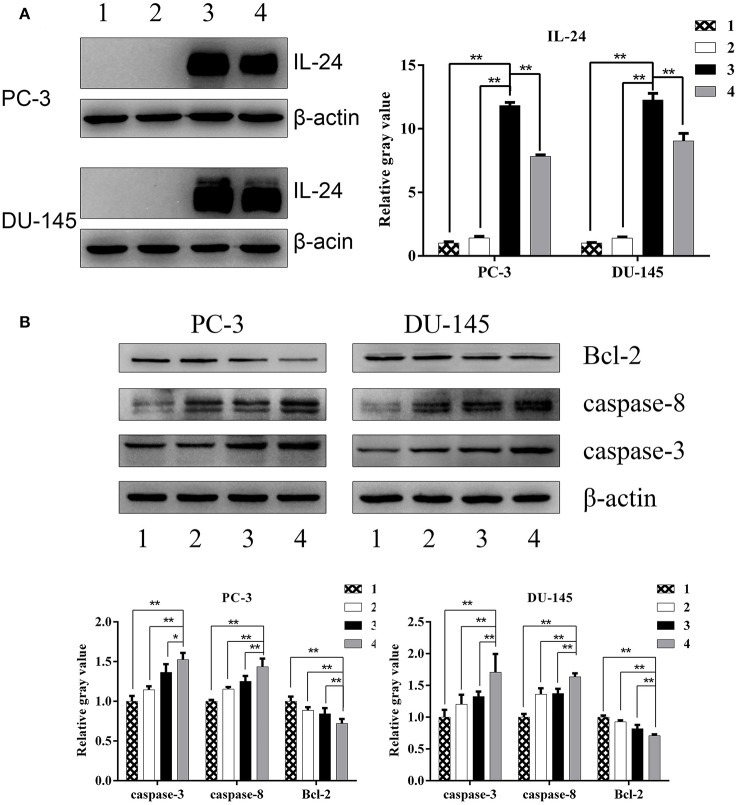
The expression of apoptosis related proteins in PC-3 and DU-145 cells exposed to ZD55-IL-24 or/and radiation. **(A)** Western blot analysis of IL-24 protein levels in PC-3 and DU-145 cells in each group. **(B)** Western blot analysis of Bcl-2, caspase-3, and caspase-8 protein levels in PC-3 and DU-145 cells in each group. 1, PBS; 2, radiation; 3, ZD55-IL-24; 4, combination of ZD55-IL-24 and radiation. *P < 0.05, **P < 0.01 vs. combination group.

Western blot analysis showed high expression of IL-24 in PCa cells treated by ZD55-IL-24 (Figure 3A, *P* < 0.01). The protein levels of Caspase-3 and Caspase-8 in combination group were significantly higher than in monotherapy group (Figure 3B, *P* < 0.05), while Bcl-2 protein levels in combination group were significantly lower than in monotherapy group (Figure 3B, *P* < 0.01). These results indicated that ZD55-IL-24 and radiation modulated the expression of apoptosis related proteins.

### Combination of ZD55-IL24 and Radiation Inhibited Xenograft Tumor Growth in Nude Mice

**Figure 4:**
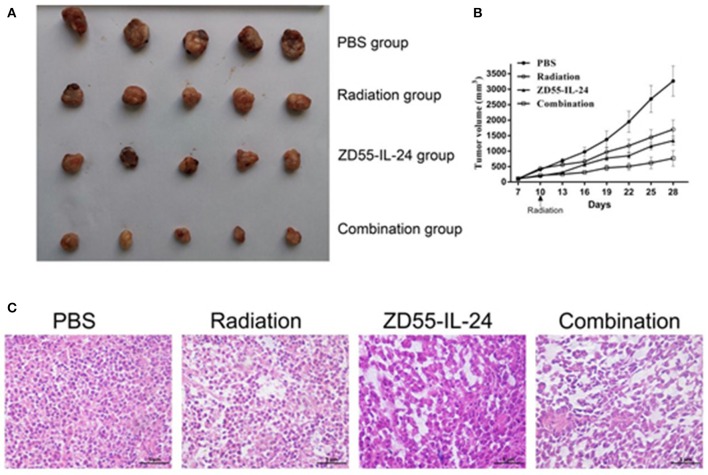
Combination of ZD55-IL-24 and radiation inhibited the growth of xenografts. **(A)** Prostate tumor xenografts were harvested from each group. **(B)** Tumor volume was measured at 3-day intervals. Date were expressed as tumor volume ± SD (*n* = 5). Tumor growth curves were drawn to show the growth of tumors in each group. **(C)** HE staining of xenografts of different groups (400×). 1, PBS; 2, radiation; 3, ZD55-IL-24; 4, combination of ZD55-IL-24 and radiation.

Next we examined the synergistic anti-tumor effects of ZD55-IL24 and radiation *in vivo*. The time-growth curve of xenografts showed the final volumes of xenografts in each group as follows: combination group: (768.56 ± 251.61) mm^3^; ZD55-IL-24 group: (1338.87 ± 143.60) mm^3^, radiation group: (1701.68 ± 297.79) mm^3^, PBS group (3265.03 ± 489.72) mm^3^. Compared with ZD55-IL-24 group, radiation group and PBS group, combination group could significantly inhibit the growth of xenografts (Figures 4A,B, *P* < 0.01). Furthermore, HE staining showed that tumor cells in PBS group had different size nuclei, and had irregular shape. In contrast, combination group showed more dead cells that split into pieces with fractured nucleus pyknosis (Figure 4C).

### Combination of ZD55-IL24 and Radiation Induced Xenograft Tumor Apoptosis

**Figure 5:**
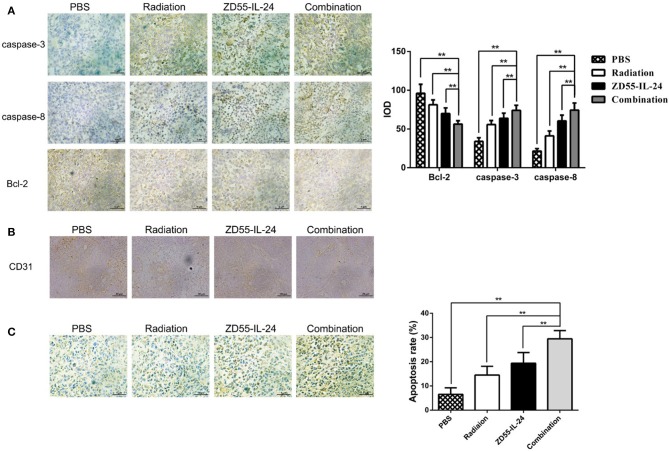
Combination of ZD55-IL-24 and radiation induced the apoptosis of xenografts. **(A)** Immunohistochemistry staining of Bcl-2, caspase-3, and caspase-8 in xenografts of different groups (400×). **(B)** CD31 staining of xenografts in each group of mice. **(C)** TUNEL staining of apoptotic cells in xenografts of different groups (400×). 1, PBS; 2, radiation; 3, ZD55-IL-24; 4, combination of ZD55-IL-24 and radiation. **P < 0.01 vs. combination group.

Immunohistochemistry analysis of xenografts showed that the integrated optical density (IOD) of Bcl-2 in combination group, ZD55-IL-24 group, radiation group and PBS group was (56.26 ± 4.46), (69.93 ± 7.33), (81.36 ± 6.18), (96.11 ± 11.56), respectively. Compared with ZD55-IL-24 group, radiation group and PBS group, combination group could significantly downregulate Bcl-2 expression (Figure 5A, *P* < 0.01). The IOD of caspase-3 in combination group, ZD55-IL-24 group, radiation group and PBS group was (34.11 ± 4.65), (55.84 ± 5.07), (63.77 ± 6.69), (74.02 ± 6.69), respectively. Compared with ZD55-IL-24 group, radiation group and PBS group, combination group could significantly upregulate caspase-3 expression (Figure 5A, *P* < 0.01). The IOD of Caspase-8 in combination group, ZD55-IL-24 group, radiation group and PBS group was (21.59 ± 3.08), (41.14 ± 6.27), (60.48 ± 7.51), (74.43 ± 9.17), respectively. Compared with ZD55-IL-24 group, radiation group and PBS group, combination group could significantly upregulate caspase-8 expression (Figure 5A, *P* < 0.01). Furthermore, combination group showed weaker CD31 staining than monotherapy group (Figure 5B).

TUNEL assay indicated that the apoptosis rate of combination group, ZD55-IL-24 group, radiation group and PBS group in tumor tissues was (6.56 ± 2.70), (14.53 ± 3.58), (19.38 ± 4.41), (29.50 ± 3.36)%, respectively, with significant difference between the combination treatment group and the monotherapy group (Figure 5C, *P* < 0.01).

**Figure 6:**
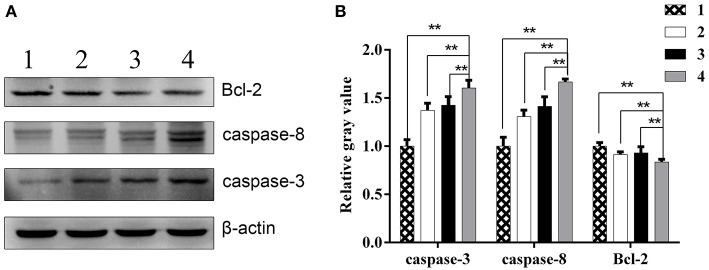
The expression of apoptosis related proteins in xenografts. **(A)** Western blot analysis of Bcl-2, caspase-3, and caspase-8 protein levels in xenografts of different groups. **(B)** Quantitative analysis of relative Bcl-2, caspase-3, and caspase-8 protein levels in xenografts of different groups. 1, PBS; 2, radiation; 3, ZD55-IL-24; 4, combination of ZD55-IL-24 and radiation. **P < 0.01 vs. combination group.

Moreover, Western blot analysis showed that relative Bcl-2 protein levels in combination group, ZD55-IL-24 group, radiation group and PBS group were (0.84 ± 0.03), (0.93 ± 0.06), (0.91 ± 0.03), (1.00 ± 0.04), respectively. Compared with ZD55-IL-24 group, radiation group and PBS group, combination group significantly downregulated Bcl-2 expression (Figure 6, *P* < 0.01). Relative Caspase-3 protein levels in combination group, ZD55-IL-24 group, radiation group and PBS group were (1.60 ± 0.08), (1.43 ± 0.09), (1.37 ± 0.07), (1.00 ± 0.07), respectively. Compared with ZD55-IL-24 group, radiation group and PBS group, combination group significantly upregulated Caspase-3 expression (Figure 6, *P* < 0.01). Relative Caspase-8 protein levels in combination group, ZD55-IL-24 group, radiation group and PBS group were (1.67 ± 0.03), (1.41 ± 0.10), (1.31 ± 0.07), (1.00 ± 0.09), respectively. Compared with ZD55-IL-24 group, radiation group and PBS group, combination group significantly upregulated Caspase-8 expression (Figure 6, *P* < 0.01). Collectively, these results demonstrated that combination of ZD55-IL-24 and radiation had better apoptosis-inducing capability *in vivo*.

## Discussion

The sensitivity of tumors to ionizing radiation and drugs depends on gene expression in the cells. The abnormal expression of oncogenes and tumor suppressors would influence tumor radiosensitivity. The overexpression of HER-2/neu gene is associated with the resistance of tumor cells to radiation therapy (11). The mutation of p53 gene is also related to tumor resistance to radiotherapy (12, 13). In addition, IL-24 could enhance the sensitivity of tumors to radiotherapy (14, 15). How to enhance the radiosensitivity of tumor cells and the effectiveness of radiotherapy has become a challenge.

Oncolytic virotherapy is a promising treatment for tumors. CTGVT showed better anti-tumor effect. ZD55-IL-24 can inhibit the growth of tumors and express IL-24 in cancer cells. ZD55-IL-24 is better than single oncolytic virotherapy and gene therapy. Therefore, we wondered whether ZD55-IL-24 could further increase radiosensitivity of prostate cancer. In this study, we aimed to investigate the anti-tumor effect of combining ZD55-IL-24 with radiation therapy in prostate cancer. We found that the combination of ZD55-IL-24 and ionizing radiation exhibited better inhibition effect on prostate cancer cell viability and better induction of prostate cancer cell apoptosis *in vitro*. These results suggest that the combined therapy strategy is feasible. To explore the anti-tumor effects of combined therapy *in vivo*, we constructed a xenograft tumor model in nude mouse. Based on this *in vivo* model, we confirmed that the combined therapy had stronger inhibition on the growth of xenograft tumor than single virotherapy and radiation therapy.

The induction of cell apoptosis plays a major role in anti-tumor mechanism. Proteins of cysteine-aspartic acid protease (caspase) family, inhibitor of apoptosis proteins (IAPs) family and B-cell lymphoma-2 (Bcl-2) family are implicated in the regulation of apoptosis (16–18). Caspase-3, caspase-8 and caspase-9 belong to caspase family and participate in the initiation and execution of apoptosis (19). Caspase-9 is involved in mitochondrial mediated endogenous pathway of apoptosis, while Caspase-8 is involved in death receptor mediated exogenous pathway of apoptosis. Caspase-3 is a downstream effector protein that leads a cascade reaction after it is activated (19). In both *in vitro* and *in vivo* experiments, we found that all the therapy increased the levels of caspase-3 and caspase-8 but decreased the level of anti-apoptotic Bcl-2. These changes were more significant in combined therapy than other single therapy. Furthermore, the results of TUNEL assay demonstrated that the combined therapy induced stronger cell apoptosis.

CD31 (platelet endothelial cell adhesion molecule-1, PECAM-1) is expressed in blood vessels and lymphatic endothelial cells and involved in tumor angiogenesis and metastasis (20, 21). The combination group showed weaker CD31 expression compared to single therapy. These results suggest that ZD55-IL-24 combined with radiation could inhibit the angiogenesis of prostate cancer. Further studies are needed to investigate the underlying mechanism.

In conclusion, the combination of ionizing radiation and oncolytic adenovirus expressing IL24 could achieve synergistic anti-tumor effect on prostate cancer, and is a promising strategy for prostate cancer therapy.

## Data Availability Statement

The raw data supporting the conclusions of this article will be made available by the authors, without undue reservation.

## Author Contributions

LM, YK, BL, SM, YL, and DY performed the experiments. CY designed the study. All authors read and approved the manuscript.

### Conflict of Interest

The authors declare that the research was conducted in the absence of any commercial or financial relationships that could be construed as a potential conflict of interest.
